# Collaborative Learning Among Health Care Organizations to Improve Quality and Advance Racial Equity

**DOI:** 10.1089/heq.2023.0098

**Published:** 2023-09-13

**Authors:** Ivan A. Copado, Amanda L. Brewster, Sarah D. Epstein, Timothy T. Brown, Hector P. Rodriguez

**Affiliations:** Division of Health Policy and Management, School of Public Health, University of California, Berkeley, California, USA.

**Keywords:** learning collaboratives, quality improvement, racial disparities, coaching, health equity

## Abstract

**Background:**

The study examined stakeholder experiences of a statewide learning collaborative, sponsored and led by Blue Cross Blue Shield of Massachusetts (BCBSMA) and facilitated by the Institute for Healthcare Improvement (IHI) to reduce racial and ethnic disparities in quality of care.

**Methods:**

Interviews of key stakeholders (*n*=44) were analyzed to assess experiences of collaborative learning and interventions to reduce racial and ethnic disparities in quality of care. The interviews included BCBSMA, IHI, provider groups, and external experts.

**Results:**

Breast cancer screening, colorectal cancer screening, hypertension management, and diabetes management were focal areas for reducing disparities. Collaborative learning methods involved expert coaching, group meetings, and sharing of best practices. Interventions tested included pharmacist-led medication management, strategies to improve the collection of race, ethnicity, and language (REaL) data, transportation access improvement, and community health worker approaches. Stakeholder experiences highlighted three themes: (1) the learning collaborative enabled the testing of interventions by provider groups, (2) infrastructure and pilot funding were foundational investments, but groups needed more resources than they initially anticipated, and (3) expertise in quality improvement and health equity were critical for the testing of interventions and groups anticipated needing this expertise into the future.

**Conclusions:**

BCBSMA's learning collaborative and intervention funding supported contracted providers in enhancing REaL data collection, implementing equity-focused interventions on a small scale, and evaluating their feasibility and impact. The collaborative facilitated learning among groups on innovative approaches for reducing racial disparities in quality. Concerns about sustainability underscore the importance of expertise for implementing initiatives to reduce racial and ethnic disparities.

## Background

The COVID-19 pandemic exacerbated racial and ethnic disparities in the quality of care in the United States, resulting in increased public awareness and organizational efforts to advance health equity. Blue Cross Blue Shield of Massachusetts (BCBSMA), a large statewide insurer, is making progress in reducing disparities in quality by adopting collaborative learning methods developed by the Institute for Healthcare Improvement (IHI) and implementing payment reforms that incentivize reductions in racial inequities.^1,2^

Learning collaboratives allow health care organizations to create teams with the goal of using pedagogical resources to achieve similar objectives. These objectives include implementing cost-effective methods, improving patient care, enhancing health outcomes, promoting patient interactions, and facilitating data sharing.^3–7^ Results of collaborative efforts across studies differ, with some indicating improvements, whereas others show no differential changes.^4–10^ However, implementation of collaborative learning systems, such as the IHI Breakthrough Series (BTS), can lead to improvements, including long-term implementation of plans designed during sessions.^4–6,11^ The BTS convenes expert faculty and quality improvement (QI) teams for workshops aimed at learning, designing, and implementing techniques to bridge gaps in care.^2–6^ Groups then implement interventions through Plan-Do-Study-Act (PDSA) cycles.^2–6^

There is limited information about collaborative learning systems that aim to reduce racial disparities in quality of care. In 2021, BCBSMA granted over $25 million to aid contracted provider groups' efforts to reduce racial/ethnic disparities in quality of care.^1,2^ Provider groups invited to participate were Alternative Quality Contract (AQC) provider groups. Provider groups using the AQC model reduced spending rates by 12% between over 8 years, while also improving their Health Care Effectiveness Data and Information Set (HEDIS) measures.^12,13^ Incentives for reducing racial disparities were not used during this time. The racial justice movement, which gained momentum due to the disparities exposed by the pandemic and was further intensified by the murder of George Floyd, stimulated health care organizations' efforts to become anti-racist institutions.^14–17^

As a result of the AQC's impact and the racial justice movement, BCBSMA is beginning to revise their AQC contracts to include incentives for racial equity improvement in select HEDIS measures, which can account for 20% of their total incentive pay.^12^ AQC groups were also invited to participate in the Equity Action Committee (EAC), BCBSMA's learning collaborative, to develop and test interventions that addressed racial/ethnic disparities in quality of care identified within their own electronic health record data and BCBSMA's claims data.^1^ With funding from BCBSMA, IHI facilitated the equity improvement collaborative of teams from 12 AQC groups to disseminate best practices and to test and assess the impact of pilot projects aimed at reducing racial/ethnic disparities in quality.

Our study examines stakeholder experiences of the EAC. We analyzed the use of pilot grant funds and stakeholder experiences of identifying quality disparities and testing interventions to reduce racial and ethnic disparities. The qualitative research study makes a new contribution to evidence about learning collaboratives by examining how collaborative processes impact the development of strategies aimed at reducing quality of care disparities. Our findings can provide an important foundation for future insurer-led efforts for provider groups aimed at advancing racial equity.

## Methods

### Data and study design

A qualitative interview design was used to investigate participant experiences of BCBSMA's equity initiative. A qualitative approach allowed us to gain a comprehensive understanding of stakeholder perspectives through their own words, providing us with detailed data about implementation. Between October 12, 2022 and March 16, 2023, we reached out to 60 individuals and conducted 42 interviews of 44 individuals involved with or impacted by BCBSMA's equity initiatives (Table 1).

**Table 1.** Key Stakeholders of Blue Cross Blue Shield of Massachusetts' Equity Initiative, by Group

Group	Attempted	Completed
All stakeholders	60 (100%)	44 (73.3%)
BCBSMA leadership and staff	18 (30.0%)	16 (26.7%)^a^
External stakeholders	5 (8.3%)	3 (5.0%)^a^
Provider groups	15 (25.0%)	10 (16.7%)
Health equity council	9 (15.0%)	5 (8.3%)
Institute for health care improvement	9 (15.0%)	7 (11.7%)
Small provider groups	4 (6.7%)	1 (1.7%)
Individual stakeholder roles^a^		
Executive	43 (71.7%)	30 (50.0%)
Manager	4 (6.7%)	3 (5.0%)
Physician	16 (26.7%)	11 (18.3%)
Analyst/research	6 (10.0%)	6 (10.0%)
Coach	5 (8.3%)	4 (6.7%)

^a^ Some key stakeholders held multiple roles.

BCBSMA, Blue Cross Blue Shield of Massachusetts.

Researchers developed and finalized interview questions that assessed stakeholder roles, stakeholder familiarity with BCBSMA equity initiative components, efforts to improve race, ethnicity, and language (REaL) data collection, EAC experiences, and the use of infrastructure and pilot grant funding. Guided by past research on strategies to advance health equity,^18^ a codebook of 11 codes and 16 subcodes was iteratively developed by four researchers. Participants reviewed a consent document before their 30- to 60-min recorded interviews, which were transcribed and coded after de-identification.

### Analyses

These analyses examined stakeholder experiences of the EAC and infrastructure and pilot grant implementation components of BCBSMA equity initiatives. We used deductive/inductive analysis methods to identify primary themes shared by stakeholders regarding the EAC process, infrastructure grants, and pilot project grants. Researchers used NVivo LLC software to code and analyze the transcripts. To ensure reliability, multiple researchers coded 10 interviews spanning the stakeholder groups, which were reviewed by four researchers to reach consensus. The 33 remaining transcripts were coded by one of the four researchers. Coding practices were reconciled and emerging themes were identified during weekly team meetings. NVivo analysis features were used to examine all text segments associated with focal codes, and themes were documented. Supporting text and quotes were organized within these themes.

## Results

### QI priorities

BCBSMA provided groups with their 2019 and 2020 HEDIS performance data, stratified by race/ethnicity as well infrastructure grants and pilot grant resources. The stratified data stimulated several provider groups to identify quality gaps to assess quality of care disparities using data from their own clinical/administrative information systems for the first time. Provider groups' pilot projects targeted quality measures with high potential for reducing racial and ethnic disparities, including breast cancer screening, colorectal cancer screening, hypertension management, and type 2 diabetes care management. Groups implemented targeted interventions, including pharmacist medication management, mobile clinics for hypertension, and home health visits. Black/African American and Hispanic/Latino patients were found to have significant quality of care disparities for most provider groups. As a result, most groups' interventions were targeted for these patient populations.

### Learning collaborative experiences

Our analyses of EAC activities revealed three key themes: (1) the EAC enabled the testing of interventions by provider groups; (2) infrastructure and pilot funding were important, but groups needed more resources than they initially anticipated; and (3) expertise in QI and health equity was critical for testing interventions. See Table 2 for an overview of all themes and corresponding quotes and text.

**Table 2.** Equity Action Community Themes and Illustrative Quotes

The IHI collaborative process as a key component of the initiative, enabling the 12 provider groups to interact and formulate plans together	“[IHI sessions are] a great convening of others towards a similar goal… In order to drive this type of transformative change, I think you have to have something like this.” –AQC group Executive and provider “BCBSMA… has definitely spurred the continued focus on equity… as opposed to… our own internal work that had been happening before.” – AQC group provider “Since we're very new to this space, we want to be thoughtful of how we approached it.” – AQC group provider
BCBSMA grant funds, administered by IHI through the learning collaborative, motivated provider group participation, and facilitated external input, but the AQC groups found that implementing equity-based interventions required more resources and effort than anticipated after receiving the grants.	“Teams initially [received] $250,000 to participate in just the [learning collaborative]… And now [more] money is getting out the door, teams are engaged and we've seen some good results from teams.” – BCBSMA Executive “[When] a team wants to improve diabetes care, hypertension care, or the maternal experience… they may not have data that shows a disparity between groups they serve. [This] means there is often a larger upfront period where they explore their own system for data that indicates a gap exists." – IHI Coach “We'll be looking for [ways] to keep [the] programs sustainable. And then of course, keeping a very close eye on metrics that can cost us like readmissions, ED visits, et cetera. And that can take a lot of time, but the hope is that we'd be able to reduce utilization so that when the grant is no longer funded, we'll be funding it ourselves.” – AQC group Executive and provider “It's not just the money. [BCBSMA has] also matched data capability with the provider organization's data capabilities.” – IHI Executive “[W]e did a full… bottoms up budget calculation over a five year period… And you know, the grant is 2 million…But what we guesstimated…is about a hundred million over a five year type of investment.” – AQC Stakeholder “I sat in on one meeting, where [BCBSMA and IHI stakeholders] were saying… ‘You're not gonna lose money,’ which is good at least for the first few years, but eventually you're gonna need to close these disparities in care.” – AQC group provider “And it's just a challenge because I think taking time to do those system changes… is really hard when you're already so resource strapped and time strapped. But I think that's one of the biggest areas [of] focus…how will we make these changes sustainable? And how do we not just focus on adding more but changing steps within the processes that aren't working?” – IHI Coach
Expertise in QI and health equity was considered very important to provider groups to support their testing of new interventions to reduce racial and ethnic disparities in quality of care.	“The majority of [race and ethnicity] questions… indicators are blank. So when we first pulled our data… it was limited… because of all of our systems that don't always work [share information].” – AQC group provider “Patients may ask, ‘why do you need to know that?’ We need to make sure our staff feels they have the appropriate responses to those questions.” – AQC group provider “We have the clinical meetings with them and then there's another meeting about data collection… [This] is helping people to know that [clinical interventions are] what we need to do now… And then…we meet one-on-one [to] think through… barriers, and…. to start to work on those things…. And we can come up with a solution to a [clinical problem] and pilot that solution to get things done.” – IHI Coach “We are in the process of deploying the resources that Blue Cross and IHI provided to us, targeting diabetes interventions. And having the support of a consultant to discuss what we're doing in the PDSA cycles and doing process mapping and so on has been helpful while we wait to start [the pilot].” – AQC group provider

AQC, Alternative Quality Contract; ED, Emergency Department; IHI, Institute for Healthcare Improvement; PDSA, Plan-Do-Study-Act cycles; QI, quality improvement.

#### Theme 1: The EAC enabled the 12 provider groups to interact, and to formulate and implement pilot interventions that addressed racial disparities in quality of care

Stakeholders indicated that the EAC was integral to BCBSMA's equity initiative, because it offered expert coaching and a platform for provider groups to share ideas. Participants in the initiative utilized IHI-designed learning processes, including PDSAs, to expose potential flaws and successes. Monthly meetings and workshops with IHI coaches enabled participants to improve upon their respective interventions, yielding results beyond what stakeholders could have achieved individually. For instance, an AQC group executive stated,

“[EAC sessions are] a great convening of others towards a similar goal… In order to drive this type of transformative change, I think you have to have something like this.”

The EAC emphasized group learning, which was particularly valuable for organizations in the early stages of their own equity-focused efforts. The community aspect provided a forum for newer groups to thoughtfully design their initiatives, as noted by a smaller AQC group provider. Larger AQC groups tended to have pre-established equity-focused initiatives, driven by Chief Equity Officers or other executives, commonly with limited funding. As a result, their internal initiatives often took longer to design and implement than those designed for the EAC. As one AQC group provider observed,

“BCBSMA… has definitely spurred the continued focus on equity… as opposed to… our own internal work that had been happening before.”

The EAC contributed to ongoing efforts to promote equity within provider groups, building on prior efforts or offering to groups without their own equity initiatives a means to prioritize an equity-focus. By providing a platform for AQC groups, regardless of their size or experience, the EAC enabled groups to make progress in this critical area. Through an iterative process based on IHI's approach, the AQC groups have developed methods for achieving targeted equity improvements on prioritized HEDIS measures.^2,3^

#### Theme 2: BCBSMA grant funds, administered by IHI through the EAC, motivated AQC group participation, but implementing equity-focused interventions required more resources than they initially anticipated

As of March 2023, BCBSMA had allocated $25 million in funding to support group infrastructure and the IHI-facilitated pilot grants. Each AQC group was invited to participate in the EAC, which provided expert coaching, monthly meetings, and sharing of best practices about approaches to advance racial health equity. As noted by a BCBSMA executive,

“Teams initially [received] $250,000 to participate in just the [EAC]… And now [pilot grant] money is getting out the door, teams are engaged and we've seen some good results from teams.”

Provider group engagement in the EAC led to information sharing and the identification of shared quality of care priorities among the provider groups to allow IHI to provide more support and resources that could benefit all, such as REaL data collection protocols and evidence-based interventions for managing chronic conditions and improving the provision of preventive care. BCBSMA provided infrastructure support to complement funding allocation efforts. As noted by an IHI executive,

“It's not just the money. [BCBSMA has] also matched data capability with the provider organization's data capabilities.”

A BCBSMA executive explained that the extra funding has assisted provider groups with hiring more clinical support staff to help with data collection, community outreach, and mobile outreach efforts. These efforts have resulted in substantial improvement in REaL data for AQC group provider groups. Some groups did not fully understand their quality of care disparities by race and ethnicity before engaging in the EAC. Some provider groups previously lacked REaL data, but with BCBSMA's infrastructural support, they could now standardly collect and routinely access REaL data from their patients. Driven by REaL data collection processes refined through the EAC and additional infrastructure support from BCBSMA-sponsored grants, AQC groups identified geographic areas and practice sites with concentrated disparities. An IHI coach said:

“[When] a team wants to improve diabetes care, hypertension care, or the maternal experience… they may not have data that shows a disparity between groups they serve. [This] means there is often a larger upfront period where they explore their own system for data that indicates a gap exists.”

AQC groups were encouraged by BCBSMA to use a payer-agnostic approach to their infrastructure investments and pilot grant activities. Interventions were designed to have the most impact on the group's total patient population for a prioritized HEDIS measure regardless of the BCBSMA-specific patient counts for the measure. Thus, AQC groups were encouraged to focus on their total patient population with the assistance from expert coaches and peers in the EAC. BCBSMA leadership understood that their competitors' patients would benefit from these investments, but believed that it was more important for groups to build their infrastructure and experience with addressing racial disparities than worry about the potential free-riding of competitor health plans.

While AQC group leaders considered the infrastructure and pilot grant funding provided by BCBSMA as foundational investments, some raised concerns about the sustainability of engaging in QI by race/ethnicity for their organizations. These concerns were shared during EAC meetings, as shared by an IHI coach:

“And it's just a challenge because I think taking time to do those system changes… is really hard when you're already so resource strapped and time strapped. But I think that's one of the biggest areas [of] focus…how will we make these changes sustainable? And how do we not just focus on adding more but changing steps within the processes that aren't working?”

Key stakeholders stressed that continued funding for collaborative learning and interventions would be important to sustain the equity-focused interventions they implemented as part of the EAC.

#### Theme 3: Expertise in QI and health equity were considered very important to provider groups to support their testing of new interventions to reduce racial and ethnic disparities in quality of care

AQC group stakeholders raised concerns about patient responses to requests for self-reported REaL data. AQC group providers stressed the importance of adequate staff training for REaL data collection during IHI coaching sessions, including how to communicate the purpose of collecting race and ethnicity data and how to address patients' concerns. Although coaches understood AQC group challenges with REaL data collection, they often had to redirect efforts toward clinical interventions, relying on BCBSMA data to guide improvement. One IHI coach stated,

“We have the clinical meetings with them and then…another meeting about data collection… [This] is helping people to know that [clinical interventions are] what we need to do now… And then…we meet one-on-one [to] think through… barriers, and…start to work on those things…. And we can come up with a solution to a [clinical problem] and pilot that solution to get things done.”

Some AQC group providers wanted to delay implementation of pilot interventions until after BCBSMA grant funding had been fully dispersed and coaching interactions were complete. Although understandable, groups often needed to be reminded that delays could limit the benefits of interventions for racial and ethnic minority patients and that implementing the pilot would not negatively impact their operations. As one AQC group provider highlighted, consulting with coaches led to focused use of resources:

“We are in the process of deploying the resources that BCBSMA and IHI provided to us, targeting diabetes interventions. And having the support of a consultant to discuss what we're doing in the PDSA cycles and doing process mapping and so on has been helpful while we wait to start [the pilot].”

AQC group stakeholders consistently identified access to expert coaches as catalytic in enabling them to address racial and ethnic disparities in quality of care. As one IHI executive described the process,

“The faculty…offer once a month coaching calls… that's when they're really able to [learn] how they can apply different change ideas and use the model for improvement to improve equity and care.”

IHI coaches had expertise in QI and interventions to address racial and ethnic disparities in care. With their guidance, AQC groups were able to identify populations to target for interventions, assess intervention impacts on quality disparities, and refine their interventions.

## Discussion

BCBSMA's equity initiative is one of the first U.S. statewide payer efforts to advance racial health equity through payment reform and collaborative learning. The collaborative learning methods used in the EAC, including expert coaching and group meetings helped groups measure and begin to address racial and ethnic disparities in quality of care. Interventions developed and tested during the pilot program included pharmacist-led medication management, REaL data collection improvement, mobile clinics, community health worker approaches, and strategies to improve transportation availability.

Our analysis found both facilitators and challenges of addressing racial and ethnic disparities through the EAC: (1) the EAC enabled the testing of interventions by provider groups; (2) infrastructure and pilot funding were important, but groups needed more resources than they initially anticipated; and (3) expertise in QI and health equity were critical for the testing of interventions. The identified themes provide insights into the facilitators and challenges of using learning collaboratives in QI efforts to address racial and ethnic disparities.

Coaches with expertise in addressing racial and ethnic disparities enabled AQC groups, some of whom were previously unfamiliar with implementing interventions for Black, Latino, and other vulnerable populations, to approach the initiative with greater ease and develop effective interventions within the EAC. In addition, coaches helped the groups identify the widest quality disparities and target interventions, which were generally Black/African American and/or Hispanic/Latino populations. This allowed groups to focus their improvement work. In addition, rapport between coaches and the teams enabled coaches to encourage provider groups to implement interventions and overcome initial hesitation among some team members.

Coaches also helped the groups identify evidence-based interventions for vulnerable populations, including mobile clinics, community-based outreach, and patient navigation by community health workers to address quality disparities. Consistent with past evidence, coaches were an important source of social support and project management expertise, which facilitated continuous QI efforts for the groups.^19,4–6^ Teams were guided through PDSA cycles by the coaches, where interventions were tested on a small scale for efficacy and feasibility.^4–6^

Our findings can help insurers and provider groups begin to tackle racial and ethnic disparities in quality of care. The pilot grant funding was a central resource for this initiative. Stakeholders indicated that continued funding for interventions to reduce racial and ethnic disparities would be needed, otherwise the BCBSMA investments could unravel.^4–6^ BCBSMA contracted IHI to oversee the pilot grant fund allocation process, encouraging provider groups to actively engage in the IHI facilitated collaborative learning process. The collaborative learning system allowed for multiple provider groups to efficiently design and test clinical interventions.

The findings should be considered in light of some limitations. First, although our response rate was high at 73%, nonparticipants may have had different experiences that may provide insights about the challenges of collaborative learning.^20^ Second, multiple informants per AQC group represented could have enabled an assessment of consistency of experiences within groups. Future research should examine heterogeneity of member experiences within provider groups.

## Conclusions

Our interviews of stakeholders of the EAC aimed at advancing health equity highlighted the value of peer learning and coaching. The EAC supported groups in their efforts to improve REaL data collection and to test interventions on a small scale to assess their feasibility and impact on advancing racial equity. BCBSMA grant funding enabled provider groups to thoughtfully design and implement interventions, such as pharmacist medication management, increasing clinic transportation availability, and implementing community health worker approaches.

Concerns about sustainability of equity-focused interventions tested as part of the EAC, continued access to coaches, and availability of patient-reported data, however, were common concerns among provider groups. Learning collaboratives have the potential to reduce the overall cost of QI because of the ability to import best practices.^8,4–6^ The results underscore the importance of payer alignment, so that provider groups can continue to advance their organizational initiatives to reduce racial and ethnic disparities.

## Abbreviations Used

AQC: Alternative Quality Contract
BCBSMA: Blue Cross Blue Shield of Massachusetts
BTS: Breakthrough Series
EAC: Equity Action Committee
ED: Emergency Department
HEDIS: Health Care Effectiveness Data and Information Set
IHI: Institute for Healthcare Improvement
PDSA: Plan-Do-Study-Act
QI: quality improvement
REaL: race, ethnicity, and language
